# Charge-Density-Wave Control by Adatom Manipulation and Its Effect on Magnetic Nanostructures

**DOI:** 10.1021/acs.nanolett.4c04581

**Published:** 2024-12-19

**Authors:** Lisa M. Rütten, Eva Liebhaber, Kai Rossnagel, Katharina J. Franke

**Affiliations:** † Fachbereich Physik, Freie Universität Berlin, 14195 Berlin, Germany; ‡ Institut für Experimentelle und Angewandte Physik, Christian-Albrechts-Universität zu Kiel, 24098 Kiel, Germany; ¶ Ruprecht Haensel Laboratory, Deutsches Elektronen-Synchrotron DESY, 22607 Hamburg, Germany

**Keywords:** charge-density wave, niobium diselenide, Yu−Shiba−Rusinov states, superconductivity, scanning tunneling microscopy, atom manipulation

## Abstract

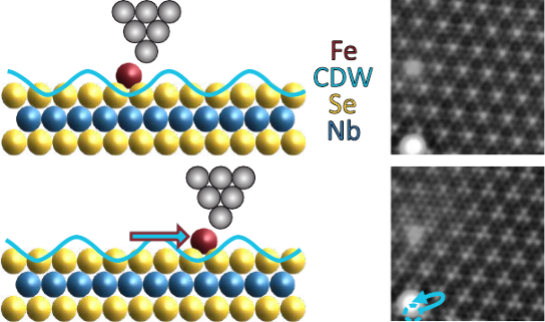

Charge-density waves (CDWs) are correlated states of matter, in which the electronic density is modulated periodically due to electronic and phononic interactions. Often, CDW phases coexist with other correlated states, such as superconductivity, spin-density waves, or Mott insulators. Controlling CDW phases may, therefore, enable the manipulation of the energy landscape of these interacting states. The transition metal dichalcogenide 2*H*-NbSe_2_ hosts both CDW order and superconductivity, with the incommensurate CDW phase resulting in different CDW-to-lattice alignments at the atomic scale. Using scanning tunneling microscopy, we position adatoms on the surface to induce reversible CDW domain switching. We show that the domain structure critically affects other local interactions, particularly the hybridization of Yu–Shiba–Rusinov states, which emerge from exchange interactions of magnetic Fe atoms with the superconductor. Our results suggest that CDW manipulation could also be used to introduce domain walls into coupled spin chains on superconductors, potentially impacting topological superconductivity.

Correlated states of matter are among the most widely studied phenomena in modern solid-state physics. When correlated states of matter are combined within the same material, their competition is particularly intriguing and gives rise to a rich phase diagram.^1,2^ The competition demands a deep understanding of the relevant energy scales and at the same time opens pathways for tuning the balance of the coexisting phases, possibly also driving phase transitions.^3^ Among the interesting correlated states, superconductivity and charge-density order play particularly prominent roles.

2*H*-NbSe_2_ is a prime example of a material that exhibits both charge-density waves (CDW) and superconductivity at low temperatures. In this material, superconductivity is of the Bardeen–Cooper–Schrieffer type albeit with a highly anisotropic Fermi surface leading to a complicated superconducting gap structure.^4−9^ After intense studies, the origin of the CDW has been attributed to strong momentum-dependent electron–phonon interactions and the softening of phonon modes at low temperatures rather than Fermi surface nesting driving the transition to the charge-density ordered phase.^10−12^

Additional interest in CDW in 2*H*-NbSe_2_ arises from its incommensurability with the atomic lattice. Different alignments with respect to the lattice have varying energies,^13^ causing the long-range order to fragment into domains separated by topological domain walls.^14^ Defects additionally influence the energy landscape of the CDW alignment. Thus, they may effectively act as pinning centers of the CDW’s domains^15−22^ or even serve as seeds in materials that are close to the phase transition, such as the sister compound 2*H*-NbS_2_.^23^ Additionally, a recent study using terahertz pulses coupled into a scanning tunneling microscopy (STM) junction showed that defects play a crucial role in the dynamics of CDW excitations.^24^ Defect engineering is thus a highly promising avenue for controlling charge-density order and possibly even building resonators for CDW excitations.

However, while defects are a natural choice for manipulation of CDW phases, bulk defect engineering imparts several disadvantages. First, it is not easy to control their location during the growth process, second, they may affect other material properties, and third, it is impossible to change their position in the bulk a posteriori. Hence, alternative approaches for manipulating CDWs have been explored. One suggestion entails the application of strain.^17,25,26^ However, it is very difficult to control and determine the strain on the atomic scale. In another avenue, it was shown that lateral voltage pulses applied to a thin flake induced changes in the CDW order.^27^ This method is restricted to thin samples equipped with additional gates. Voltage pulses in the junction of a scanning tunneling microscope have also successfully induced changes in CDW phases.^28−31^ However, this process is statistical in nature, thus imposing challenges on the precise control and reversibility of the switching.

Here, we suggest combining the advantages of defect engineering with the atomic-scale precision of STM. Individual atoms on the surface act as defects that can be positioned at will by dragging them with the STM tip. While we show that adatoms may be used for controlling the domain of the structure of the CDW, we take advantage of a second important role of adatoms. When using magnetic atoms on a superconducting CDW material, their exchange-scattering potential causes Yu–Shiba–Rusinov (YSR) states inside the superconducting gap of the substrate.^32−36^ Interestingly, the energy of the YSR states and their wave function’s symmetry are influenced by the CDW due to the spatial variation of the density of states at the Fermi level.^37^ The presence of the CDW may thus be a curse and a blessing at the same time. Because of the variation of the YSR energy along the charge-density modulation, the hybridization properties of closely spaced atoms may be changed if the CDW is switched. It has been shown that the energy of the YSR bands in atomic chains extending across CDW domain boundaries varies in the different domains.^38^ However, this appeared to be a property that could not be manipulated in a controlled manner. Here, we demonstrate the potential of manipulating the CDW alignment to reversibly change the YSR hybridization properties at the example of an Fe dimer adsorbed on 2*H*-NbSe_2_ by positioning other adatoms around the dimer.

STM images of the 2*H*-NbSe_2_ substrate reveal the atomic structure of the terminating Se layer with an additional apparent-height modulation with a lattice constant of *a*_CDW_ > 3*a* (with *a* being the Se atomic lattice constant) reflecting the CDW (Figure 1a–e). Its incommensurate nature translates into a variation of the alignment of the CDW maxima along the atomic lattice. We show a large-scale topographic image revealing this variation along several domains in the Supporting Information (Figure S1). In the case of the CDW maximum coinciding with a Se atom (schematic in Figure 1f), we observe a hexagonal pattern as shown in the close-up in Figure 1d. We refer to this alignment as chalcogen-centered (CC). Contrasting this alignment is a hollow-centered (HC) area, which resembles a three-petal pattern, as shown in a close-up in Figure 1e and sketched in Figure 1g. We indicate the CC and HC CDW areas in Figure 1a–c with blue and orange shading, respectively. The transition between these domains has been assigned to topological domain walls.^14^

**Figure 1:**
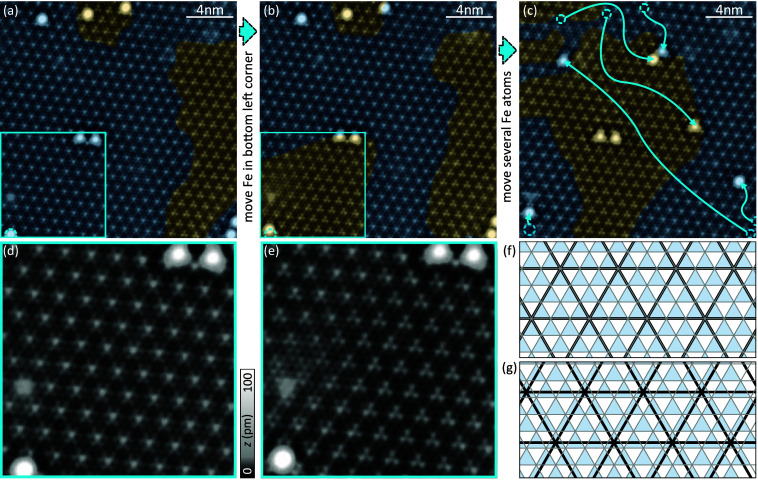
Manipulation of the CDW. (a–c) Three stages of manipulating the CDW around an Fe dimer from CC to HC by lateral manipulation of Fe adatoms. CC and HC CDW areas are colored blue and orange, respectively. In panel c, the positions of the Fe atoms prior to manipulation are marked by circles while the arrow marks their final position. (d) Close-up of the CC CDW region of the blue square in panel a. (e) Close-up of the same region shown in panel d after the CDW was switched to HC in this area. (f and g) Schematic of the CC and HC CDW-to-lattice alignment, respectively, where gray lines indicate the Se lattice, black lines represent the CDW (maxima at crossings), and hollow sites are colored blue. Set point in panels a–e: 10 mV, 50 pA.

After deposition at low temperature, we observe two kinds of Fe atoms on the surface (see Note 2 of the Supporting Information) that differ in apparent height and shape. These types correspond to different adsorption sites with respect to the crystal lattice: The larger, round adatoms are adsorbed in hollow sites of the Se lattice, albeit with a Nb atom underneath. This site has been termed the metal site (MS). The smaller, triangular-shaped adatoms are located in hollow sites among three Se atoms without an atom below, which is therefore termed the hollow site (HS).^37,39^ Both atoms in the center of Figure 1a–c are located in hollow sites with respect to the Se lattice. The analysis of the adsorption site is found in Note 3 of the Supporting Information. They will remain in their precise position in the following and used to probe the effect of a change of the CDW alignment as explained below.

To manipulate the CDW alignment, we first move the Fe atom in the bottom left corner closer to the bright defect above it, as indicated by the blue dashed circle that marks the initial position of the atom in panels a and b of Figure 1. The CDW pattern in the bottom left quadrant (indicated by the blue squares in panels a and b of Figure 1 and magnified in panels d and e of Figure 1) then changes from its hexagonal shape signaling CC alignment (Figure 1a,d) to the three-petal shape of the HC alignment (Figure 1b,e). Note that even the bright defect within this area changes its appearance from hexagonal to triangular. The drastic change caused by a small variation in the position of a single Fe atom is rather surprising and indicates energetically closely laid CDW-to-lattice arrangements. Next, we also move other Fe atoms located close to the edges of our scan frame in panels a and b of Figure 1. A topographic image of the resulting arrangement is presented in Figure 1c, where arrows indicate the original positions of all atoms in panel b. The CDW now assumes the HC configuration in the center of the scan frame (i.e., around the HC Fe dimer). Images of individual manipulation steps are shown in Note 8 of the Supporting Information. The switching of the CDW-to-lattice arrangement can be reversed as we show in Note 7 of the Supporting Information.

The possibility of changing the CDW domain structure by single atoms is in line with the expectation that defects alter the energy landscape of the CDW alignment. However, we note that each change in the position of a single atom does not necessarily lead to a rearrangement of the CDW phase. Rather, it is the energy landscape imposed by the distribution of adatoms that dictates the final ground state. Our technique of CDW manipulation thus benefits from a fine energy balance of all atomic potentials in the vicinity. In turn, an assembly of several adatoms may be used to stabilize larger areas of a certain CDW alignment, within which one can freely manipulate single atoms without affecting the CDW. This option is important for realizing larger adatom structures with stable properties. Conversely, one may take advantage of the capability to change the CDW and thereby change the properties of the adatom structures. In the following, we show this at the example of the Fe dimer in Figure 1. In particular, we want to highlight how the CDW switch leads to a change in the properties of hybridized YSR states.

To fully appreciate the influence of the CDW on the coupled YSR states of an Fe dimer, we need to briefly introduce the YSR states of a single Fe atom and their hybridization characteristics. Figure 2a shows a topographic image of an isolated Fe atom. This atom is the very same as the right atom of the dimer in Figure 1a before the left atom was brought into its vicinity. It is adsorbed in a hollow site next to a CDW minimum of the CC alignment. (For a detailed view of the atomic-site determination, see Note 2 of the Supporting Information.) The differential conductance (d*I*/d*V*) spectrum taken on the atom’s center is displayed in Figure 2b (black) together with a spectrum of the bare substrate (gray). The Fe spectrum shows multiple YSR states labeled α, β, γ, and δ in the sequence of their energy, and in agreement with ref (37). The multiplet is a consequence of four singly occupied *d* levels that are crystal-field split and exchange coupled to the substrate.^37,40,41^ Differential conductance maps of the two deepest-lying resonances (α and β) are shown in panels c and d of Figure 2 and exhibit one mirror plane (dashed line). The reduction from the 3-fold symmetry of the atomic adsorption site to 2-fold is caused by the CDW alignment.^37^ The symmetry is visualized in Figure 2e, where the atomic hollow sites are labeled by colored triangles sitting on the background of the 3 × 3 CDW (thick black lines). The CC CDW-to-lattice alignment (thick black lines coinciding with thin gray lines of the Se lattice) preserves only one mirror plane for all possible adsorption sites. This symmetry reduction is indicated by a dashed line for the site marked by a black circle, which represents the adsorption site of the Fe atom.

**Figure 2:**
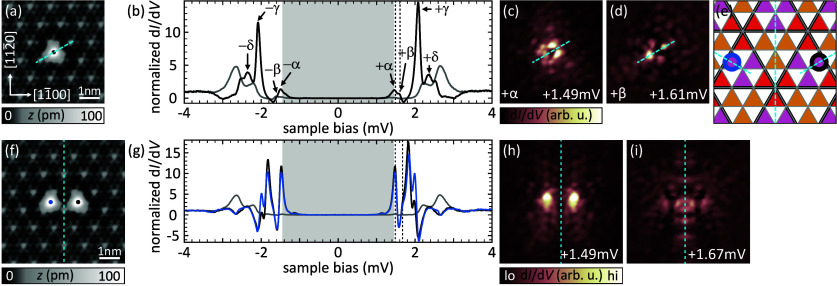
Fe monomer and dimer on the CC CDW domain. (a and f) Topographic images of an Fe atom and an Fe dimer with a spacing of 4*a*, respectively. All mirror planes are indicated by dashed lines. (b and g) d*I*/d*V* spectra recorded at the positions indicated in panels a and f, respectively. Gray traces were recorded on the bare substrate. (c, d, h, and i) Constant contour d*I*/d*V* maps recorded at the energies of the two energetically lowest resonances of the monomer (c and d) and dimer (h and i). (e) Schematic of the 2*H*-NbSe_2_ surface in a CC CDW area. Hollow sites are color coded according to their CDW position, and the adsorption sites of both atoms are indicated by color-coded circles. All mirror axes present for the individual atoms, as well as the dimer, are indicated by dashed lines. Δ_tip_ ≈ 1.44 mV. Set points for panels a and f: 10 mV, 50 pA; rest: 5 mV, 750 pA; all: *V*_rms_ = 15 μV.

Figure 2f shows a close-up topographic image of the same Fe atom now with an additional Fe atom (indicated by the blue dot) pushed into its vicinity. More precisely, the second atom is located in a hollow site of the Se lattice at a distance of four atomic lattice sites (4*a*) (for structure determination, see Note 2 of the Supporting Information). We therefore name this arrangement a 4*a* dimer. It is precisely the one at the center of the scan frame in Figure 1a–c. Both atoms of the dimer exhibit similar d*I*/d*V* spectra with an increased number of YSR states compared to the monomer (Figure 2g). A doubling of the number of resonances is expected for hybridization. While our energy resolution prohibits a clear identification of eight resonances within the small energy region of the gap, the spatial distribution of the lowest-lying resonances corroborates the interpretation of hybridized YSR states. We show the corresponding d*I*/d*V* maps in panels h and i of Figure 2. The maps feature a nodal plane (Figure 2h) and a maximum intensity (Figure 2i) perpendicular to the bonding axis. Therefore, we can assign them to the antisymmetric and symmetric linear combination of the monomer YSR wave functions, respectively. A detailed analysis of the adsorption site of the 4*a* dimer with respect to the CDW reveals that both atoms occupy equivalent sites. The corresponding structure including the CDW is sketched in Figure 2e with blue and black circles representing the two atoms. Overall, the structure preserves a mirror plane perpendicular to the bonding axis. As a consequence, the hybridized YSR states derived from the symmetric and antisymmetric linear combination of the monomers’ states inherit this symmetry.^38,42,43^

Next, we investigate the influence of the change of the CDW from the CC alignment to the HC alignment that was induced by manipulation, as discussed for panels b and c of Figure 1. A close-up of the dimer after the CDW switch is shown in Figure 3a with the HC CDW well resolved around the dimer. A detailed analysis of the structure ensures that the atoms did not move (see Note 2 of the Supporting Information). The d*I*/d*V* spectra recorded on the atoms changed dramatically upon the CDW switch (Figure 3b). While they still exhibit more than four YSR pairs, which is indicative of hybridization, the spectra on the two atoms now differ significantly from one another. The spectrum recorded on the right atom (black trace) shows several intense YSR states deep inside the superconducting gap, while the left one (blue trace) has more intensity close to the coherence peaks in addition to resonances deep inside the gap.

**Figure 3:**
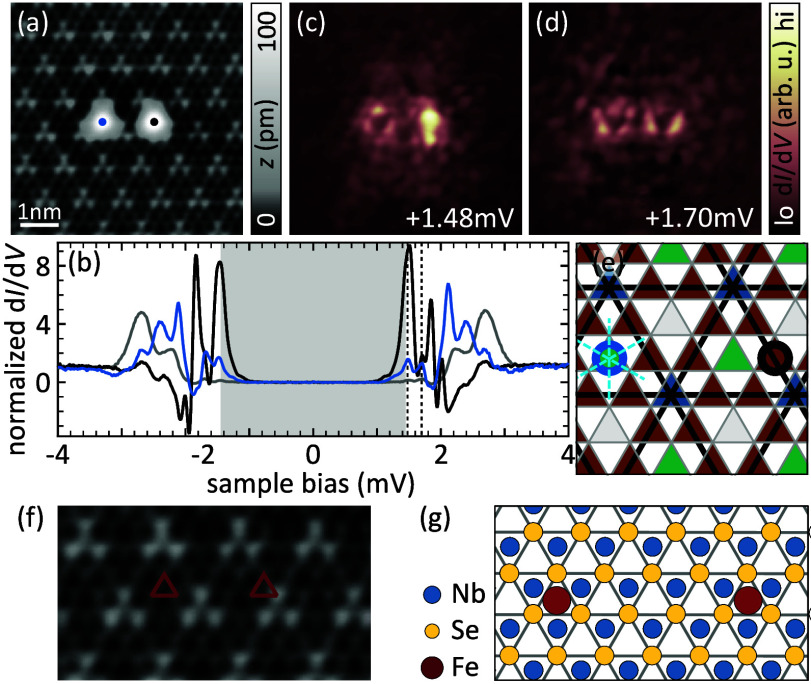
Fe dimer after switching into the HC CDW alignment. (a) Topographic image of the 4*a* dimer after switching to the HC CDW. (b) d*I*/d*V* spectra recorded on both dimer atoms (color coded) and a background trace (gray). (c and d) d*I*/d*V* maps of the two resonances lowest in energy of the 4*a* dimer after the CDW was switched. (e) Schematic of the 2*H*-NbSe_2_ surface in an HC CDW area. Hollow sites are color coded by their position with respect to the CDW (black lines), and the adsorption sites of the dimer atoms are indicated by color-coded circles. All mirror axes present for the individual atoms as well as the dimer are indicated by dashed lines. (f) Topographic image of the bare substrate, where the adsorption sites of the atoms in the 4*a* dimer are indicated by red triangles. (g) Schematic of the adsorption geometry of the 4*a* dimer with respect to the atomic lattice. Δ_tip_ ≈ 1.44 mV. Set point for panels a and f: 10 mV, 50 pA; rest: 5 mV, 750 pA; all: *V*_rms_ = 15 μV.

In agreement with the drastically different spectra, the map of the lowest-lying resonance at a positive bias voltage (Figure 3c) reflects the loss of mirror symmetry by revealing very different YSR intensities and shapes. The map of the second resonance (Figure 3d) exhibits more similar patterns around each atom and appears almost symmetric around the dimer’s center, yet there are very faint features that do not obey mirror symmetry.

To rationalize the observed spectra and their spatial distribution, we analyze the position of the atoms with respect to the CDW (for details, see Note 2 of the Supporting Information). We find that both atoms no longer reside in equivalent sites. The left atom (blue spectrum) sits in a hollow site in a CDW minimum (sketch in Figure 3e). At this position, all three mirror axes of the Se lattice and CDW align, and the symmetry of the crystal is preserved. On the contrary, the right atom (black spectrum) is located in a hollow site on a line connecting two CDW maxima. At this position, all mirror symmetries are broken owing to the position with respect to the CDW (rather than by the underlying lattice as visible from the ball model in Figure 3g). This symmetry breaking is visible in Figure 3f, where we depict a topographic image of the pristine surface in an HC CDW area and indicate the sites of the atoms in the 4*a* dimer by red triangles. As a consequence of the different symmetries of the adsorption sites of the individual atoms, there is no mirror axis present in the dimer.

Analyzing peak positions in the d*I*/d*V* spectra of the dimer shows that the spectra cannot simply be reproduced by adding the spectra of the isolated atoms in the adsorptions sites corresponding to those in the dimer (albeit with different intensities accounting for their spatial decay). Hence, the increase in the number of peaks must originate from hybridization of the inequivalent atoms. Such hybridization patterns are distinct from those that can be obtained in gas-phase molecules because the underlying lattice adds symmetry properties that can be exploited for wave function design.^43^ To generalize this response to CDW switching, we also investigated a 2*a* dimer. The observations can also be explained by the different CDW alignments before and after a purposeful switch of the CDW. The results are shown in Note 4 of the Supporting Information.

In conclusion, controlling the position of adatoms on a CDW material is a viable route for manipulating the CDW properties. This method can be fine-tuned to obtain energy landscapes that either stabilize larger domains or are on the edge of unstable behavior, allowing for reversible switching. We highlight the potential of the effect of CDW manipulation by showing the drastic changes in YSR hybridization in Fe dimers on 2*H*-NbSe_2_. In previous experiments, the limited size of the CDW domains imposed constraints on the length of a homogeneous YSR band structure.^38^ Chains consisting of more than 11 Fe atoms extended across different domains of CDW-to-lattice alignments, where CDW-induced variations of the YSR bands were observed.^38^ The findings presented here suggest that CDW manipulation may be used to manipulate the domain structure below the YSR chains or lattices. Furthermore, CDW manipulation may change not only the energy of hybridized states but also their symmetry. In ref (38), the distance between the adatoms was chosen to be three atomic lattice sites (3*a*), which is close to the CDW periodicity. This ensured an overall mirror symmetry of the chain. Here, we chose dimers with 2*a* and 4*a* separation. In these cases, changes in the CDW are inevitably accompanied by changes in the symmetry of the hybridized YSR states. In addition to changes in the energy alignment and symmetry of the YSR states, the switching of the CDW may possibly also affect the magnetic structure. Furthermore, with the immense interest in topological states of adatom structures,^44−47^ one may envision controlling the topological properties as a consequence of, e.g., shifting the band alignment through the Fermi level.^48,49^

## Methods

Our experiments are carried out in a Joule-Thomson scanning tunneling microscope from Specs, working at 1.1 K under ultra-high-vacuum conditions. We obtain a clean and flat surface by carbon-tape cleaving and deposit Fe atoms directly into the scanning tunneling microscope at temperatures below 10 K. To increase the energy resolution of our experiment beyond the Fermi–Dirac limit, we pick up a Nb crystallite with a W tip, thereby making our tip apex superconducting. In addition to the gain in energy resolution, the use of a superconducting tip shifts all spectral features by the superconducting gap of the tip. This superconducting tip gap is indicated by the gray area in all spectra. The Nb also has ideal properties to facilitate lateral manipulation of the Fe atoms.

## Data Availability

The original experimental data are available at https://zenodo.org/records/14062244.

## Supporting Information Available

Charge-density-wave characterization, adsorption-site determination, spectral characteristics of the Fe monomer, data of the 2*a* dimer, and CDW switching and reversibility (PDF)
