# Supplementary material for: Charge-Density-Wave Control by Adatom Manipulation and Its Effect on Magnetic Nanostructures
Source: Nano Lett. 2024 Dec 19;25(1):115–20. doi: 10.1021/acs.nanolett.4c04581 (PMC11719633; doi:10.1021/acs.nanolett.4c04581)
Supplement: Supplementary file 1 — nl4c04581_si_001.pdf [file nl4c04581_si_001.pdf]

Supporting Information
Charge-density-wave control by adatom manipulation and its effect on magnetic nanostructures

Lisa M. Rütten,[†] Eva Liebhaber,[†] Kai Rossnagel,[‡] and Katharina J. Franke[*,†]

†*Fachbereich Physik, Freie Universität Berlin, 14195 Berlin, Germany*

‡*Institut für Experimentelle und Angewandte Physik, Christian-Albrechts-Universität zu Kiel, 24098 Kiel, Germany*

¶*Ruprecht Haensel Laboratory, Deutsches Elektronen-Synchrotron DESY, 22607 Hamburg, Germany*

E-mail: franke@physik.fu-berlin.de

Supporting Note 1: Charge-density-wave landscape

Cleavage of $2H$-$NbSe_2$ leads to large terraces that can be imaged with atomic resolution using STM. Figure S1 shows a 70 nm×70 nm image of the $2H$-$NbSe_2$ surface after the deposition of single Fe atoms. The Fe atoms appear as bright protrusions with apparent height >100 pm, which will be discussed in Supporting Note 2.

Notably on this large scale, we observe brighter and darker regions on the surface. We encircle the darker regions by blue dashed lines in the bottom part of the image. We associate the bright and dark regions to different domains of the charge-density wave (CDW) as we can deduce from the atomic resolution. The brighter areas correspond to domains, where

the CDW maxima coincide with Se atoms of the terminating layer. Thus, these are called chalcogen-centered (CC) CDW domains as described in more detail in the main text. The darker regions correspond to a hollow-centered (HC) CDW alignment. The overall brighter perception of CC areas can be explained by the larger average apparent height when the CDW and Se atoms are in registry.

The HC CDW regions appear to have arbitrary shape and size, while all CC CDW regions are connected in agreement with Ref. 1. If the transitions between CC and HC CDW alignment were purely governed by long-range incommensurability of the CDW, we would expect a periodic landscape of both alignments. The image thus confirms the relevance of local potentials associated to intrinsic defects and adatoms for the CDW-to-lattice alignment.

Supporting Note 2: As-deposited Fe atoms

The as-deposited Fe atoms appear as two different types, which can be distinguished by their apparent height. Figure S2b shows two atoms with the upper one (marked by a gray circle) appearing as nearly-round protrusion with an apparent height of approximately 150 pm (line profile along the arrow in Fig. S2d) while the bottom one (marked by a pink circle) appears more triangular and has an apparent height of approximately 110 pm.

To determine the adsorption sites of both atoms, we overlay a grid representing the Se lattice to the topographic image (Fig. S2c). We find that both atoms are adsorbed in hollow sites with respect to this lattice. However, the surrounding Se atoms form a triangle pointing downwards around the upper atom and a triangle pointing upwards around the lower one. Referring to a schematic top view of the $2H$-$NbSe_2$ surface as shown in Fig. S2a we can identify one of the sites as a metal site (Nb atom underneath) and the other one as a hollow site. We can determine which of the sites is a metal and which is a hollow site by looking at the CDW on the left side of the image in Fig. S2b, where the CDW assumes HC alignment. Here, the CDW maximum has to coincide with a hollow site (CDW maxima at metal sites

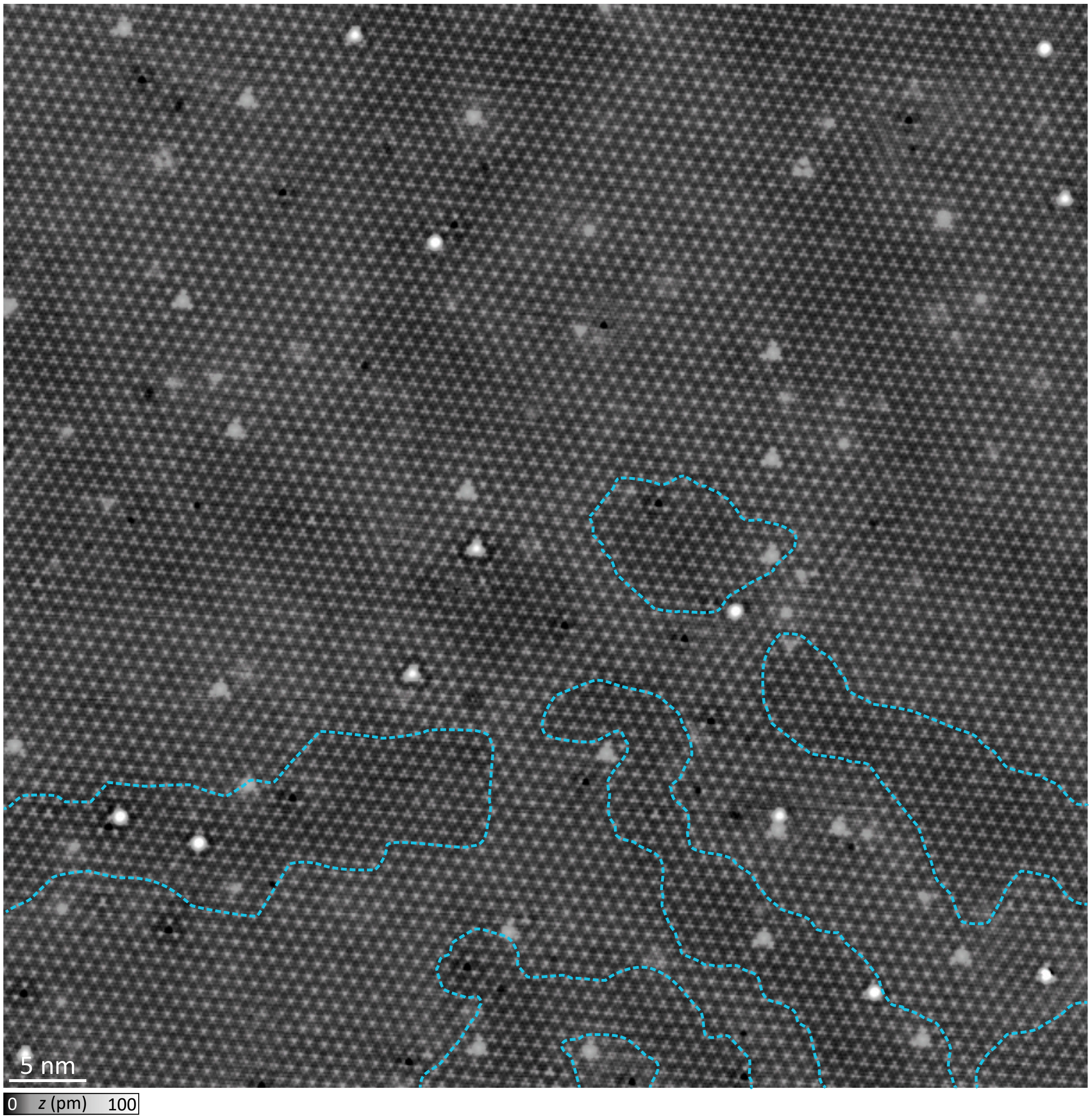

Figure S1: Overview STM image: Large-scale STM image of $2H$-$NbSe_2$ with atomic resolution after the deposition of Fe atoms. The domains of HC and CC CDW can be seen. As a guide to the eye, we surround HC CDW regions by dashed blue lines in the bottom part of the image. Set point: 10 mV, 100 pA.

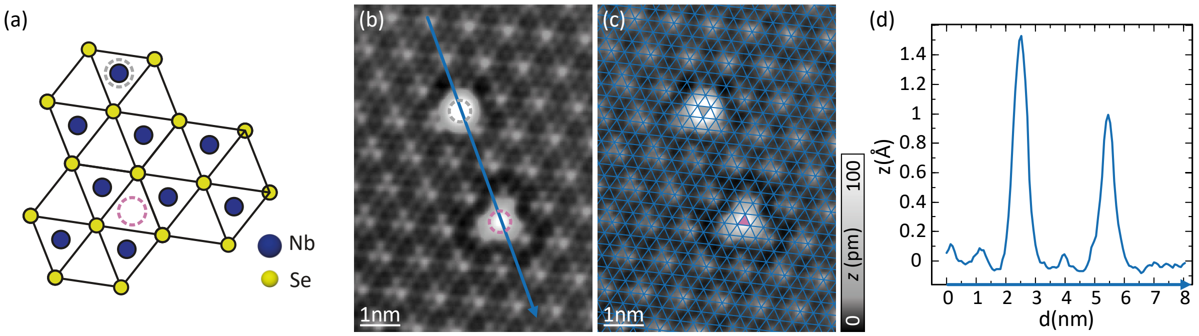

Figure S2: Types of as-deposited Fe atoms: (a) Schematic top view of a 2H-$NbSe_2$ surface with the two possible adsorption sites found in experiment indicated by color-coded circles. (b) Topographic image of two Fe atoms of different types. (c) Same image as (b) with an overlayed atomic grid of the Se atoms in the terminating layer. The associated adsorption sites are highlighted by color-coded triangles. (d) Line profile along the arrow in (b), reflecting the distinct apparent heights. Set point: (b, c) 10 mV, 50 pA.

are energetically unfavorable[1]). As the Se triangles around the CDW maxima are pointing upwards, we can conclude that sites surrounded by upward pointing Se triangles correspond to hollow sites and those surrounded by downward pointing ones correspond to metal sites. As indicated by the color-coded circles in Fig. S2a we can identify the upper (higher) atom as adsorbed in a metal site and the lower (smaller) one as adsorbed in a hollow site in agreement with previous works.[2,3]

Supporting Note 3: Determination of adsorption site

We determine the adsorption sites of the Fe atoms discussed in the main text by superimposing atomically-resolved STM images with a ball model for the $NbSe_2$ lattice and a grid of lines for the CDW. Here, yellow and blue balls correspond to selenium and niobium atoms of the topmost atomic layers, respectively, and the interceptions of the grid correspond to CDW maxima. The resulting arrangements for the Fe monomer and the dimer presented in the main text before and after the switching of the CDW are shown in Fig. S3. Here, Fig. S3a depicts the single Fe atom with the CC CDW background, (b) depicts the $4a$ dimer with the CC CDW background which has been built by adding a second atom to the left, (c)

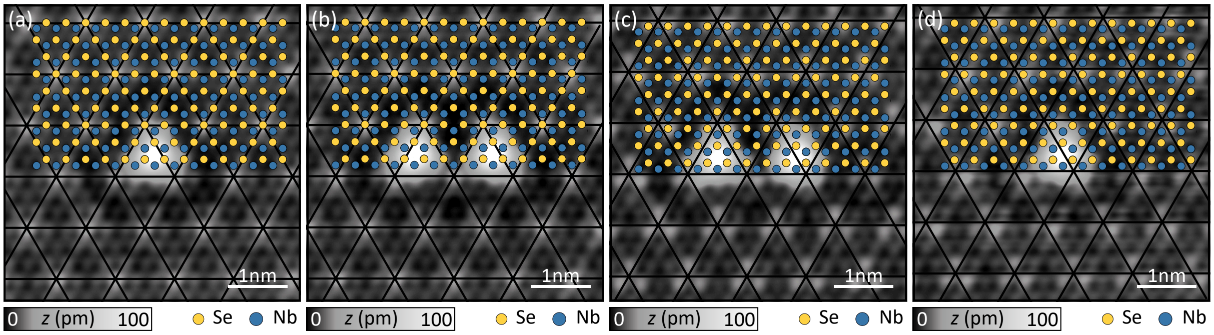

Figure S3: Adsorption-site determination: Topographic images of the monomer (a, d) and the $4a$ dimer (b, c) with the CC (a, b) and the HC (c, d) CDW background. To indicate the adsorption site we superimpose a ball model, where Nb atoms are indicated in blue and Se atoms are indicated in yellow. Additionally, the CDW is indicated by black lines where interceptions correspond to CDW maxima. Set point: 10 mV, 50 pA.

shows the same $4a$ dimer with the HC CDW background after switching of the CDW, and (d) shows the right atom after the left one has been pushed away by STM manipulation.

The analysis shows that all atoms are adsorbed in hollow sites of the atomic lattice but differ in their position with respect to the CDW. Before switching, i.e., in the CC CDW alignment, both atoms (Fig. S3a, b) reside in hollow sites next to a CDW minimum (corresponding to atom IV in Ref. 2). These sites individually exhibit one mirror axis, as described in detail in the main text. After the switching the CDW, the left atom in (Fig. S3c) resides in a hollow site at a CDW minimum (same as atom III in Ref. 2). In this position all mirror axes of CDW and atomic lattice coincide and the site is threefold symmetric. The right atom sits in a hollow site between two CDW maxima (same as atom VI in Ref. 2). In this site, no mirror axis of CDW and atomic lattice coincide, leaving the atom in an asymmetric position.

After the investigation of the influence of the CDW switch on the dimer properties, we remove the left atom by STM manipulation. The right atom then remains isolated (Fig. S3d). We note that the CDW was slightly affected by the removal of the left atom. The CDW now slips throughout the image: We find a CC CDW-to-lattice alignment in the top left half, while HC alignment is preserved in the bottom right. This change once more emphasizes the opportunity as well as limitations in the control of CDW manipulation.

Supporting Note 4: Spectral characteristics of Fe monomer after switching the CDW

We also investigate the spectroscopic signature of the monomer in Fig. S3d, i.e., after switching the CDW from CC to HC. However, as noted above, investigation of the isolated atom required removal of the left atom of the $4a$ dimer. This manipulation, unfortunately, led to a slight change of the CDW arrangement compared to the dimer as described above. The $\mathrm{d}I/\mathrm{d}V$ spectrum shows broad resonances inside the gap (Fig. S4b). Due to the limited energy resolution, we cannot identify the individual peaks originating from the crystal-field split d levels. $\mathrm{d}I/\mathrm{d}V$ maps on the energy range of the most pronounced broad peak show distinct features (Fig. S4c, d), suggesting the different d level contributions. The maps do not show any mirror symmetry in accordance with the adsorption site sketched in Fig. S4e.

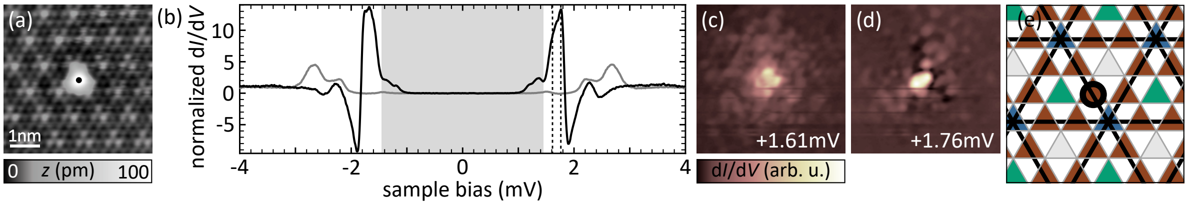

Figure S4: Spectroscopic signature of Fe monomer after CDW switch: (a) Topographic image of the Fe monomer after the CDW was switched from CC to HC. (b) $\mathrm{d}I/\mathrm{d}V$ spectra recorded at the position indicated in (a). The gray trace was recorded on the bare substrate. (c, d) Constant-contour $\mathrm{d}I/\mathrm{d}V$ maps recorded at the energies of the two energetically lowest resonances. (e) Schematic of the $NbSe_2$ surface in a HC CDW area. Hollow sites are color coded according to their CDW position and the adsorption sites of the atom is indicated by a color coded circle. In this position, all symmetries are broken. $\Delta_{\mathrm{tip}} \approx 1.44\,\mathrm{mV}$; set point: (a) 10 mV, 50 pA; rest 5 mV, 750 pA; all: $V_{\mathrm{rms}}=15\,\mu\mathrm{V}$.

Supporting Note 5: Influence of the CDW switch on a $2a$ dimer

In the main text, we demonstrate the effect of switching the CDW domain structure on a $4a$ dimer. Here, we investigate a dimer that consists of two atoms that are only two lattice

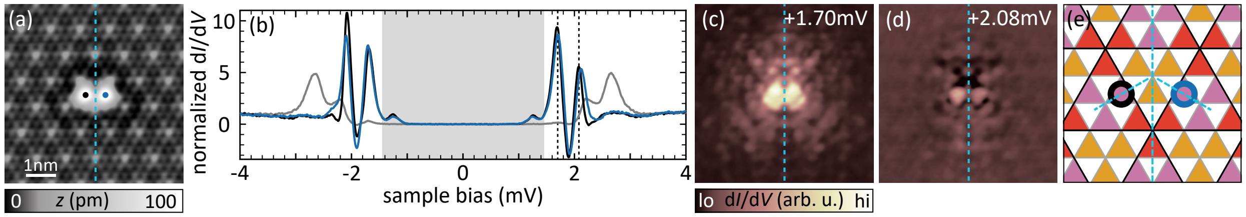

Figure S5: $2a$ dimer in CC CDW domain: (a) Topographic image of a Fe dimer with a spacing of $2a$. (b, g) $\mathrm{d}I/\mathrm{d}V$ spectra recorded at the positions indicated in (a). Gray traces were recorded on the bare substrate. (c, d) Constant contour $\mathrm{d}I/\mathrm{d}V$ maps recorded at the energies of the two energetically lowest resonances of the dimer. (e) Schematic of the $NbSe_2$ surface in a CC CDW area. Hollow sites are color coded according to their CDW position and the adsorption sites of both atoms are indicated by color coded circles. $\Delta_{\mathrm{tip}} \approx 1.44\,\mathrm{mV}$; Set point: (a) 10 mV, 50 pA; rest 5 mV, 750 pA; all: $V_{\mathrm{rms}} = 15\,\mu\mathrm{V}$.

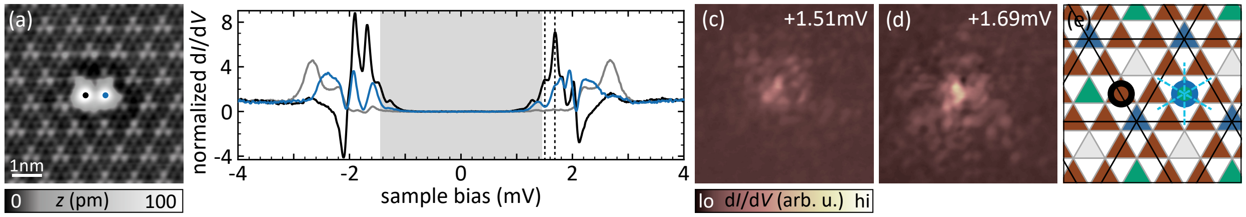

Figure S6: $2a$ dimer in HC CDW domain: (a) Topographic image of a Fe dimer with a spacing of $2a$. (b, g) $\mathrm{d}I/\mathrm{d}V$ spectra recorded at the positions indicated in (a). Gray traces were recorded on the bare substrate. (c, d) Constant contour $\mathrm{d}I/\mathrm{d}V$ maps recorded at the energies of the two energetically lowest resonances of the dimer. (e) Schematic of the $NbSe_2$ surface in a HC CDW area. Hollow sites are color coded according to their CDW position and the adsorption sites of both atoms are indicated by color coded circles. $\Delta_{\mathrm{tip}} \approx 1.44\,\mathrm{mV}$; set point: (a, f) 10 mV, 50 pA; rest 5 mV, 750 pA; all: $V_{\mathrm{rms}} = 15\,\mu\mathrm{V}$.

spacings ($2a$) apart. We note that the area is the same as the one shown in the main text and also the two atoms are the same as in the $4a$ dimer. In fact, the two dimers have been created by manipulating the two atoms (each marked by a blue and black circle for its identity, respectively) into different sites. After we recorded the data set on the $2a$ dimer we moved the atom marked by the blue circle and formed the $4a$ dimer discussed in the main text. We then switched the CDW around the $4a$ dimer and finally removed the atom marked by the blue dot to study the monomer. We then rebuilt the $2a$ dimer to be able to compare the $2a$ dimer in the two CDW domains.

The STM image of the $2a$ dimer in the CC domain is shown in Fig. S5a. Analysis of the precise adsorption sites leads to the structure sketched in Fig. S5e: Both atoms are located in hollow sites close to a CDW minimum. Each site has one mirror plane as indicated by the dashed lines. Additionally, there is a mirror plane between the two atoms. $\mathrm{d}I/\mathrm{d}V$ spectra on the two atoms are shown in Fig. S5b. The resonances are distinct from the ones seen on the single Fe atom, suggesting some interaction. However, the broad resonances prohibit a detailed analysis of the YSR hybridization. $\mathrm{d}I/\mathrm{d}V$ maps recorded at two pronounced peaks reveal the expected mirror symmetry.

After switching the CDW to the HC alignment, the $2a$ dimer was re-assembled (after the investigation of the $4a$ dimer) and is shown in Fig. S6. The $\mathrm{d}I/\mathrm{d}V$ spectra differ on the two atoms in agreement with the maps showing unequal intensity around the two atoms. The absence of any mirror symmetry in the dimer is a consequence of the adsorption sites (Fig. S6e).

Supporting Note 6: Comparison of $2a$ and $4a$ dimers in the CC and HC alignment

To bring out the changes upon switching the CDW alignment underneath the Fe monomer, $2a$ and $4a$ dimers discussed in the main manuscript and Supporting Note 5, we plot the cor-

responding spectra in Fig. S7. The gray traces are taken for reference on the bare substrate, while the blue and black spectra are recorded on the individual atoms as labeled in Fig. 2 and Fig. 3 of the main text and Fig. S5 and Fig. S6. The energies at which we recorded the dI/dV maps shown throughout the manuscript and the SI are indicated by vertical dashed lines in each spectrum. The compilation directly evidences the changes in the energies of the YSR states starting from the single atom. The shift has been explained by the variation of the density of states at the Fermi level imposed by the CDW.[2] This variation directly influences the energies of the hybridized states in the dimers. As the dimers on the CC phase were positioned with mirror symmetry between the two sites, the spectra on the individual atoms are similar. This is not the case for the same dimers in the HC phase, where the mirror symmetry is removed as described in the main text and SI Note 5.

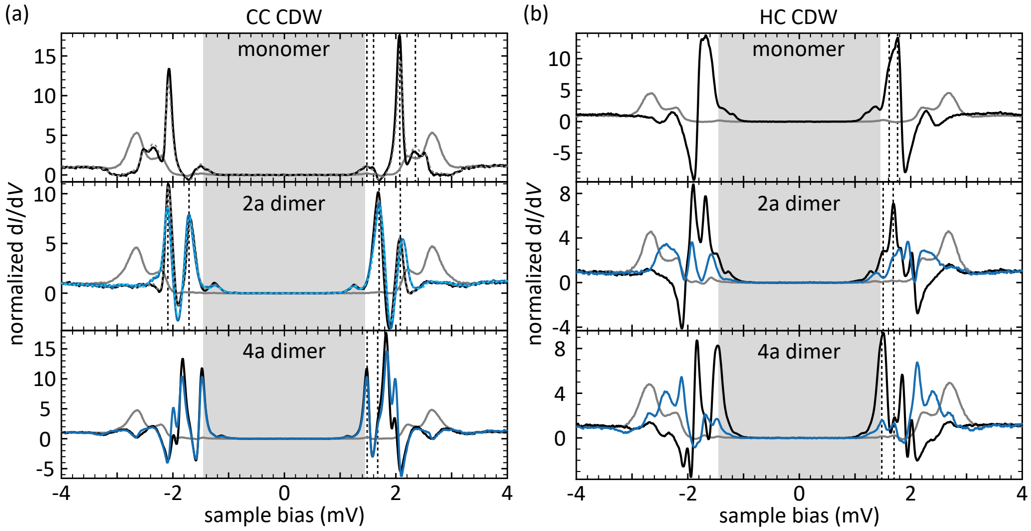

Figure S7: (a) Spectra of the monomer, 2a dimer and the 4a dimer on CC CDW alignment. (b) Spectra of the monomer, 2a dimer, and the 4a dimer on HC CDW alignment. $\Delta_{\mathrm{tip}} \approx$1.44 mV; Set point: 5 mV, 750 pA; all: V_{rms}=15 μV.

Previous experiments investigated dimers and chains with a spacing of 3a between the Fe atoms.[3] This spacing is very similar to the periodicity of the CDW. Therefore, atoms forming a 3a dimer generally reside in equivalent CDW sites. This argument holds for both CC and HC CDW alignments. In particular, the atoms forming the dimer presented in Ref. 3 reside in equivalent CDW sites in an HC CDW area, more precisely in maxima of the

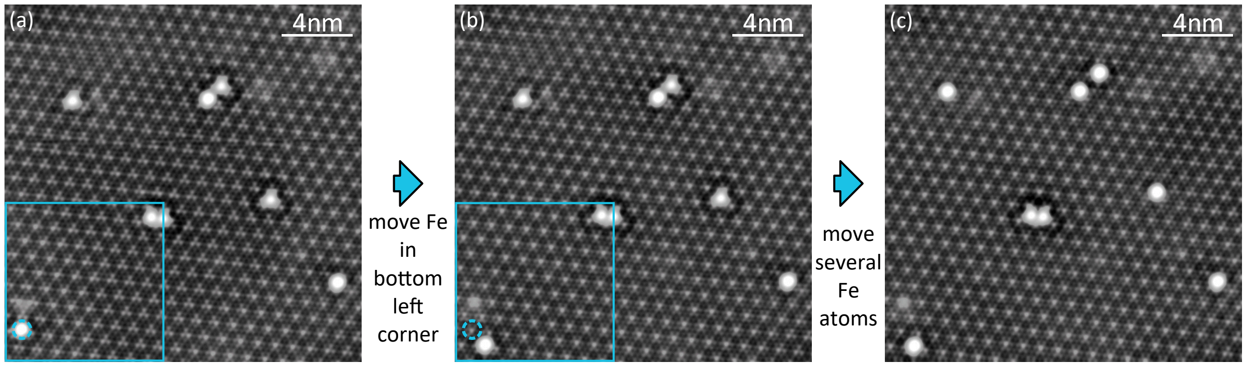

Figure S8: Manipulation of CDW around $2a$ dimer: Three stages of manipulating the CDW around the $2a$ dimer from HC to CC alignment. $\Delta_{\mathrm{tip}} \approx 1.44\,\mathrm{mV}$; set point: $10\,\mathrm{mV}$, $50\,\mathrm{pA}$.

CDW. At these sites all mirror axes of the CDW and the atomic lattice coincide. Thus, there is a mirror plane between the atoms. Therefore, hybridization in this dimer causes equivalent spectra on both atoms.

Supporting Note 7: Reversibility of CDW switching

In order to demonstrate the reversibility of the CDW switch, we once again consider the surrounding of the $2a$ dimer as shown in Fig. S8a. The central region of the scan frame (around the dimer) exhibits HC CDW-to-lattice alignment and is surrounded by CC CDW regions. We switch the CDW back to CC alignment around the dimer solely by moving the atom in the bottom left corner further away from the bright defect (Fig. S8b). This was precisely the atom that we replaced in the first step to induce the switch from CC to HC. It, thus, clearly reveals the reversibility of the switch of the CDW domain structure. Repositioning the other atoms does not change the CDW-to-lattice alignment close to the dimer as shown in Fig. S8c. We therefore assume, than one could in principle switch between both CDW to lattice alignments solely by manipulating one additional Fe atom if the other pinning Fe atoms and defects are positioned favorably.

We once again record a dataset on the $2a$ dimer after switching back the CDW to the CC domain and show it in Fig. S9a-d. We then remove the atom marked by the blue dot

and reproduce the monomer shown in Fig. 2 of the main text (Fig. S9e-h). Both datasets perfectly reproduce the ones shown in Fig. S5 for the $2a$ dimer and Fig. 2a-d of the main text for the monomer proving that the changes caused by manipulating the CDW are indeed fully reversible.

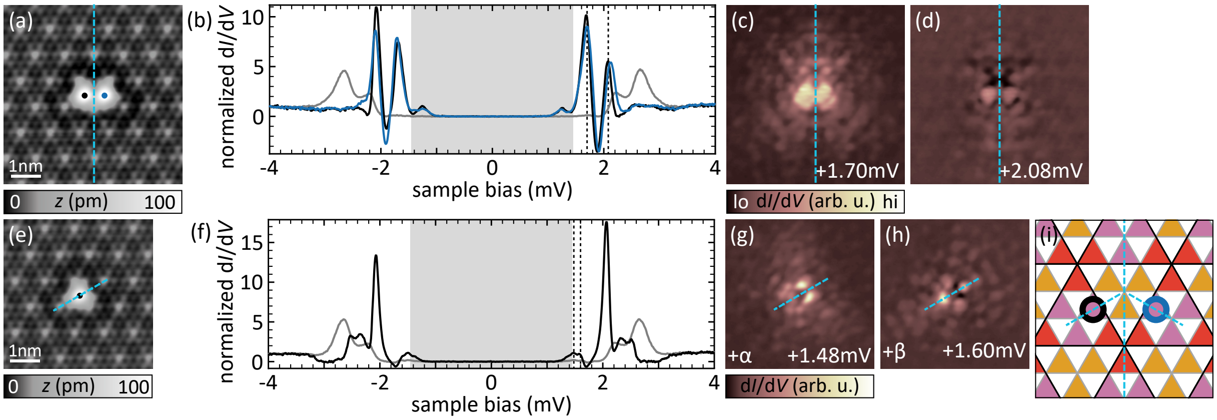

Figure S9: $2a$ dimer and monomer in CC domain: (a, e) Topographic images of the $2a$ dimer and the monomer after switching the CDW back to CC. (b, f) Spectra recorded on both dimer atoms and the monomer. (c, d, g, h) dI/dV maps of the two resonances lowest in energy of the $2a$ dimer (c, d) and the monomer (g, h) after the CDW was switched back. (i) Schematic of the $NbSe_2$ surface in an CC CDW area. Hollow sites are color coded by their position with respect to the CDW (black lines) and the adsorption sites of the dimer atoms are indicated by color coded circles. $\Delta_{tip} \approx 1.44$ mV; Set point: (a),(e) 10 mV, 100 pA; rest 5 mV, 750 pA; all: $V_{rms}=15\,\mu$V.

Supporting Note 8: Detailed data on the switching processes

Here, we show the topographic images recorded after each manipulation step for both CDW manipulations shown in the main text. We indicate which atom was moved by arrows. Figure S10 shows the manipulation of the CDW from CC to HC alignment around the $4a$ dimer. The movement of each atom is indicated by arrows in the image after manipulation of an Fe atom. The sequence of images shows that in some cases the manipulation of Fe atoms does not lead to any changes while in other cases a small displacement of a single

atom leads to a switch. The complexity of the processes reveals the delicate balance of the local minima in the energy landscape of the CDW domains.

When we manipulated the CDW back to the CC alignment (all images in Fig. S11), we needed a significantly smaller number of steps due to the understanding of the influence of the individual Fe atoms.

References

(1) Gye, G.; Oh, E.; Yeom, H. W. Topological Landscape of Competing Charge Density Waves in $2H-NbSe_2$. *Phys. Rev. Lett.* **2019**, *122*, 016403.

(2) Liebhaber, E.; Acero González, S.; Baba, R.; Reecht, G.; Heinrich, B. W.; Rohlf, S.; Rossnagel, K.; von Oppen, F.; Franke, K. J. Yu–Shiba–Rusinov States in the Charge-Density Modulated Superconductor $NbSe_2$. *Nano Lett.* **2020**, *20*, 339–344.

(3) Liebhaber, E.; Rütten, L. M.; Reecht, G.; Steiner, J. F.; Rohlf, S.; Rossnagel, K.; von Oppen, F.; Franke, K. J. Quantum spins and hybridization in artificially-constructed chains of magnetic adatoms on a superconductor. *Nat. Commun.* **2022**, *13*, 2160.

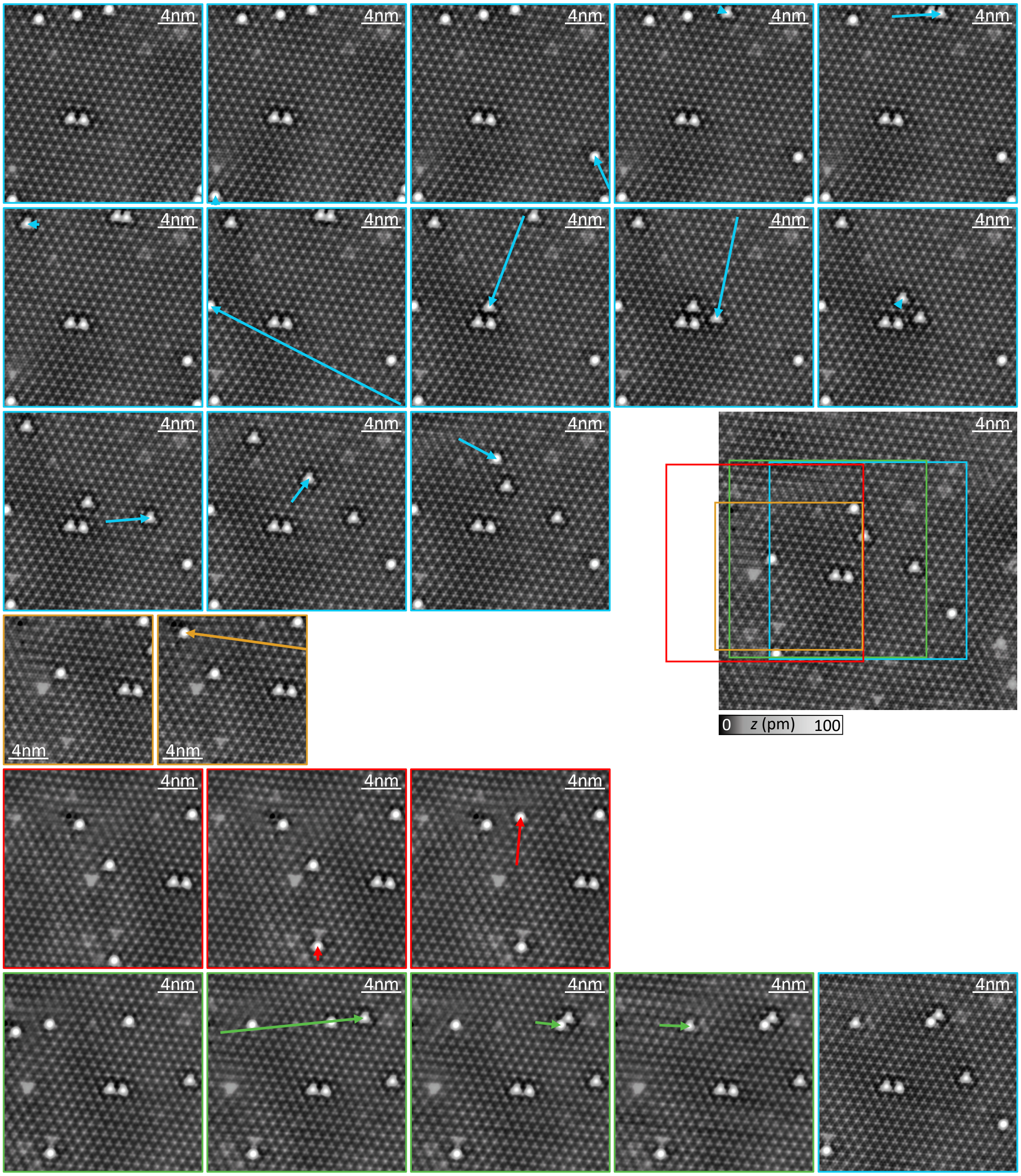

Figure S10: Topographic images of each manipulation step when manipulating the CDW from CC to HC (order left to right, top to bottom). Different scan frames are indicated by color-coded frames. Set point: 10 mV, 50 pA.

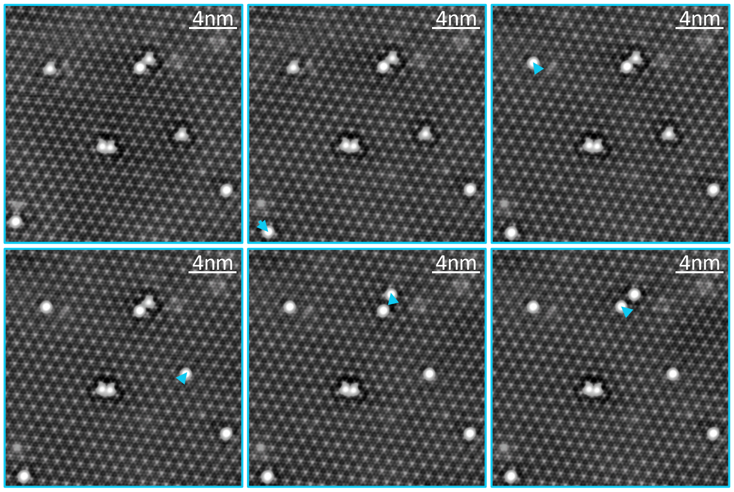

Figure S11: Topographic images of each manipulation step when manipulating the CDW back to CC alignment from left to right and top to bottom. Set point: 10 mV, 50 pA.
